# First Split Liver Transplantation Using VitaSmart Hypothermic Machine Perfusion in the United States

**DOI:** 10.1111/petr.70504

**Published:** 2026-10-15

**Authors:** Anastasia M. Kahan, Francisca van der Schyff, Todd Robinson, Ivan Rodriguez, Tavis Shaw, Enoch Zenger, M. Kyle Jensen, Jean F. Botha, Zachary J. Kastenberg

**Affiliations:** ^1^ Division of Pediatric Surgery, Department of Surgery University of Utah Salt Lake City Utah USA; ^2^ Primary Children's Hospital, Intermountain Health Salt Lake City Utah USA; ^3^ Department of Surgery, Abdominal Transplant Service Intermountain Medical Center, Intermountain Health Murray Utah USA; ^4^ Division of Gastroenterology, Hepatology, & Nutrition, Department of Pediatrics University of Utah Salt Lake City Utah USA

**Keywords:** congenital hepatic fibrosis, graft preservation, hypothermic machine perfusion, pediatric liver transplantation, split liver transplantation

## Abstract

**Background:**

Dynamic perfusion techniques are increasingly used in liver transplantation to improve graft preservation and support preimplantation assessment. Its reported use in split liver transplantation remains limited. We report the first use of the VitaSmart hypothermic perfusion device for graft preservation during ex situ split in the United States, resulting in the successful transplantation of a right trisection graft into an adult recipient and a left lateral section graft into a pediatric recipient.

**Methods:**

A deceased donor liver was split into a right trisection graft for an adult recipient and a left lateral segment graft for a pediatric recipient. Both grafts underwent VitaSmart hypothermic oxygenated perfusion. Donor, graft, perfusion, operative, and early postoperative outcomes were reviewed. The adult recipient was a 63‐year‐old man with metabolic dysfunction‐associated steatohepatitis cirrhosis. The pediatric recipient was a 4‐year‐old female with Joubert syndrome, congenital hepatic fibrosis with portal hypertension, and prior renal transplantation.

**Results:**

The right trisection graft weighed 1001 g, with an estimated graft‐to‐recipient weight ratio of 1.1. Implantation was performed with bicaval, portal vein, hepatic artery, and duct‐to‐duct biliary anastomoses. Reperfusion was uneventful, with uniform graft perfusion, normal intraoperative vascular waveforms, and bile production. The left lateral segment graft was implanted using piggyback caval implantation and Roux‐en‐Y biliary reconstruction. Postoperative Doppler demonstrated patent vessels. Early findings in both recipients demonstrated vascular patency and graft recovery.

**Conclusions:**

This initial experience demonstrates the technical feasibility of incorporating VitaSmart hypothermic machine perfusion into split liver transplantation and highlights its potential role in expanding split graft utilization.

## 1 Introduction

Split liver transplantation expands the donor pool by allowing one deceased donor liver to benefit two recipients, most commonly an adult recipient and a pediatric recipient [1, 2, 3]. Despite this advantage, split liver transplantation remains technically and logistically complex. The additional donor operative time and technical complexity of in situ splitting lead to frequent institutional bias against this technique, while the addition of relative warm ischemia time for ex situ splitting contributes to the lower survival rates, both graft and overall, observed in those receiving technical variant grafts [4, 5].

Hypothermic oxygenated machine perfusion (HOPE) has emerged as a promising strategy to improve liver graft preservation and facilitate assessment before implantation [6, 7, 8]. There are multiple benefits to HOPE, including logistical benefits related to operating room logistics, access for patients in rural or remote locations, and complex hepatectomies to allow for more controlled access. Additionally, multiple randomized control trials and meta‐analyses have shown that hypothermic oxygenated machine perfusion grafts have significantly reduced post‐reperfusion syndrome and ischemia–reperfusion injury in adult transplants compared to traditional cold preservation grafts [7, 9, 10, 11]. However, the dual inflow nature of these pumps, along with the cumbersome nature of infusion devices, while technically possible, makes graft manipulation during splitting impractical. Hypothermic perfusion, on the other hand, is generally achieved with portal inflow only, combined with open recirculation, making graft manipulation more practical. There are reports of splitting liver grafts in the setting of hypothermic machine perfusion throughout Europe, though this technique has not previously been explored in the United States [9, 12, 13, 14]. We report, to our knowledge, the first on‐pump split using the VitaSmart hypothermic perfusion device in the United States with successful transplantation of both technical variant grafts.

## 2 Case Presentation

### 2.1 Donor

The donor was a 17‐year‐old deceased brain‐death donor. The procurement was performed by a local procurement team approximately 700 miles from the transplanting centers. The graft arrived locally with approximately 8 h of cold ischemia after which it was promptly back tabled; this dissection included division of the hepatic artery and left hepatic duct leaving the portal vein and hepatic veins intact. The graft was then placed on the VitaSmart hypothermic perfusion device with portal perfusion using MPS perfusate.

The graft was perfused for 144 min with an average flow rate of 230 mL/min at a pressure of 3 mmHg. During the pump run, the parenchymal transection was performed by initially defining the transection plane along the undersurface of the liver. The ligamentum venosum was divided and the left hepatic vein was encircled. A plane was sharply developed along the lateral aspect of Segment 4 for hanging the liver with a vessel loop. The liver was then flipped, while maintaining perfusion, and the transection commenced with the hanging maneuver similar to the parenchymal transection during a living donor hepatectomy, as a guide (Figure 1). The deeper parenchymal transection was performed with the CUSA and LigaSure devices, augmented with ties and clips to secure larger portal and hepatic vein branches.

**FIGURE 1:**
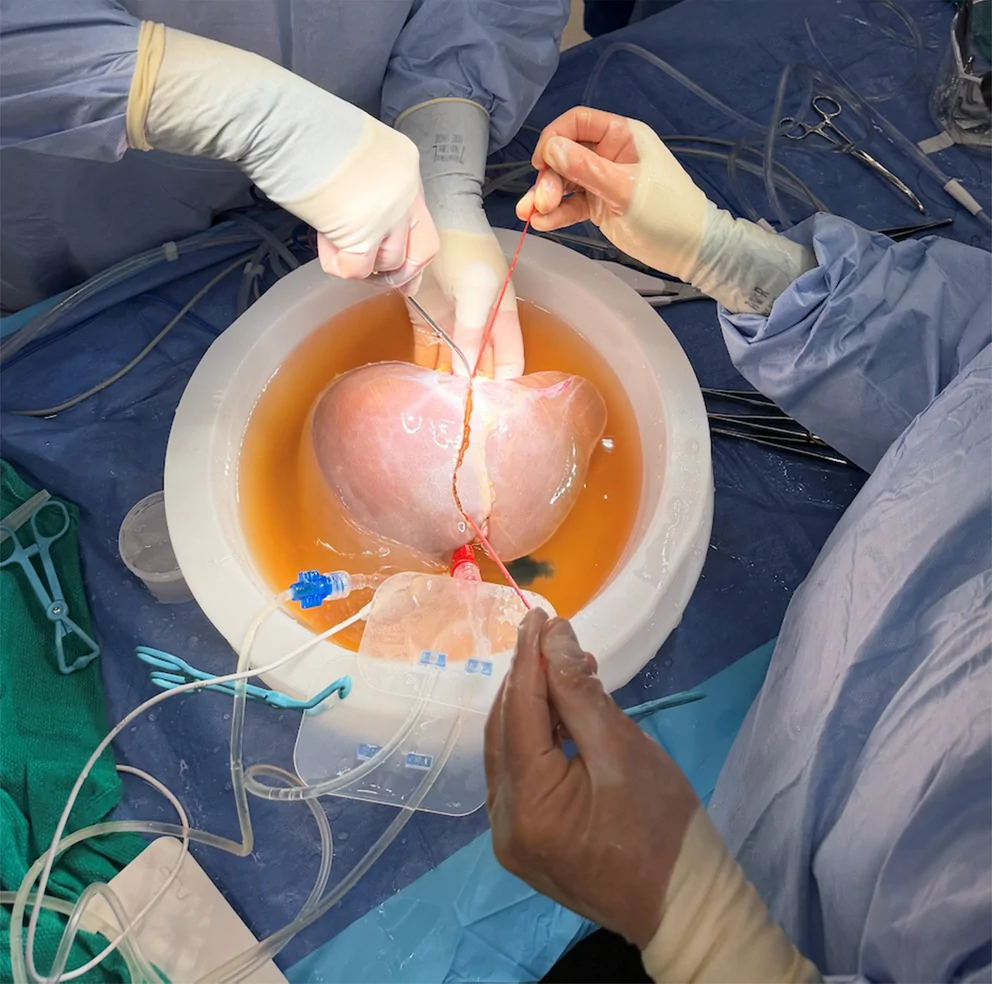
Liver graft split on the VitaSmart hypothermic perfusion pump.

After completion of the 144‐min pump run, with the hepatic veins and portal vein still in continuity, the graft was flushed with cold UW solution, the portal vein was then divided at the bifurcation leaving the main portal vein with the right trisection graft, and the left hepatic vein was sharply divided from the suprahepatic vena cava ensuring a single left vein orifice. The two grafts were then individually packaged and transported to the recipient operating rooms on ice. In this case, the decision to discontinue perfusion after completion of the split was primarily logistical. The ex‐situ split and machine perfusion were performed at the adult transplant hospital. At the completion of the split, the adult recipient hepatectomy had already been completed and the adult transplant team was prepared for immediate implantation of the right trisection graft. The pediatric recipient, however, was undergoing transplantation at a separate hospital. Therefore, following completion of the 144‐min perfusion and split, the right trisection graft proceeded to implantation, and the left lateral section graft was transported on ice to the pediatric recipient operating room at the separate hospital.

### 2.2 Right Trisection Graft

The right trisection graft recipient was a 63‐year‐old man with metabolic dysfunction‐associated steatohepatitis cirrhosis, MELD 11, and BMI 26 kg/m^2^. His primary portal hypertensive complications were diuretic‐refractory ascites and fatigue. The recipient hepatectomy was uneventful.

The right trisection graft weighed 1001 g, with an estimated graft‐to‐recipient weight ratio of 1.1%. Graft anatomy included full cava excluding the ostium of the left hepatic vein, full portal vein excluding the ostium of the left portal vein, right hepatic artery/gastroduodenal artery/common hepatic artery (the donor had a replaced left hepatic artery that went with the left lateral section graft), and full common bile duct excluding the ostium of the left hepatic duct. The left hepatic vein and left portal vein orifices were closed transversely, and the left hepatic duct orifice and two small caudate ducts were over‐sewn.

Implantation was performed in a bicaval fashion with standard arterial and portal venous reconstructions. Reperfusion was uneventful, without hemodynamic instability, and the graft reperfused uniformly. Intraoperative ultrasound demonstrated normal waveforms in all vessels, and the graft produced bile. Postoperatively, total bilirubin peaked at 8.4 mg/dL, lactate at 9.0 mmol/L, INR at 1.9, and transaminases peaked just below 1000 U/L. Bilirubin, lactate, and transaminases were all down‐trending within the first 24 h following reperfusion.

The adult recipient was discharged on PTD 5 with improving graft function (total bilirubin 2.9 mg/dL, AST 49 U/L, ALT 210 U/L, alkaline phosphatase within normal limits, creatinine 1.43 mg/dL from baseline of 1.2) and no vascular complications. Total bilirubin subsequently nadired at 1.4 mg/dL; however, evaluation for abdominal distension on PTD 13 demonstrated a 9.8‐cm fluid collection along the hepatic cut surface, labs showed an increasing alkaline phosphatase to 441 U/L. CTA demonstrated patent hepatic vasculature without arterial stenosis. Percutaneous drainage on PTD 21 yielded fluid with bilirubin > 25 mg/dL, consistent with a biloma. ERCP then performed inpatient on PT 23 demonstrated a mild biliary anastomotic stricture, and a temporary plastic stent was placed. The biloma resolved with drainage, and follow‐up CT demonstrated complete resolution of the collection. The biliary stent was removed on PTD 35, and the percutaneous drain was removed on PTD 41 after cessation of output. Liver enzymes and bilirubin subsequently normalized. A brief readmission for nausea, vomiting, and acute kidney injury (creatinine 4.0 mg/dL) was managed supportively, with renal and hepatic laboratory values returning to the patient's baseline prior to discharge.

### 2.3 Left Lateral Section Graft

The left lateral section graft was transplanted into a 4‐year‐old female with Joubert syndrome, congenital hepatic fibrosis with portal hypertension not anatomically amenable to creation of a surgical shunt. Liver biopsy demonstrated portal bridging fibrosis, and she was receiving serial endoscopic variceal ligation for Grade III varices. She had previously undergone deceased donor renal transplantation, with the renal graft positioned in the right hemiabdomen. Her weight was 14.5 kg, and her PELD was 6.

The left lateral section graft weighed 235 g, with an estimated graft‐to‐recipient weight ratio of 1.6%. Graft anatomy included a single left hepatic vein, single left portal vein, and a single replaced left hepatic artery including the left gastric artery origin divided at the celiac trifurcation. There was a single bile duct that was easily probed to confirm drainage of both segments 2 and 3. Total pump time was 144 min and total cold ischemia time was 9 h and 51 min.

Implantation included piggyback caval reconstruction, donor left portal vein to recipient main portal vein, donor left gastric artery to recipient common hepatic artery, and Roux‐en‐Y hepaticojejunostomy. The graft reperfused uniformly without systemic instability. The lactate peaked at 3 mg/dL and cleared promptly. INR peaked at 3.2 at 36 h postoperatively and then steadily down‐trended. Creatinine was maintained at preoperative levels (~0.3 mg/dL) with unaltered function of her prior kidney graft.

Postoperatively, AST and ALT peaked on postoperative day 1 at 577 U/L and 548 U/L, respectively, and declined substantially over the subsequent several days. By postoperative day 5, AST had normalized to 52 U/L and ALT had decreased to 219 U/L. Total bilirubin similarly peaked at 7.6 mg/dL on postoperative day 1 and decreased to 3.0 mg/dL by postoperative day 5, with continued improvement thereafter. GGT remained persistently elevated, increasing from 178 U/L early postoperatively to a peak of 1341 U/L one‐month post‐transplant. Given the persistent cholestatic enzyme elevation, liver biopsy was performed on post‐transplant day 45 and demonstrated focally prominent acute hepatitis with patchy, predominantly perivenular lymphocytic inflammation and mild bile duct inflammation; mild T‐cell–mediated rejection could not be excluded. CMV immunohistochemistry was negative. The decision was then made to stop sulfamethoxazole/trimethoprim and begin pentamadine for pneumocystis jirovecii pneumonia (PJP/PCP) prophylaxis and optimize tacrolimus to treat her mild rejection. At most recent follow‐up, AST was 47 U/L, ALT 54 U/L, total bilirubin 0.8 mg/dL, and GGT had decreased to 487 U/L.

## 3 Discussion

This case represents the first use of the VitaSmart hypothermic perfusion device for on‐pump splitting of a liver allograft in the United States. Both the right trisection and left lateral section grafts were successfully transplanted, with reassuring intraoperative perfusion, early rise in bilirubin and liver enzymes that declined rapidly showing early postoperative function, and early biochemical evidence of graft recovery—despite reperfusion of both grafts occurring approximately 12 h following donor cross clamp. Split liver transplantation remains frustratingly underutilized in the United States. Barriers include technical complexity, concern for graft quality, logistical coordination between recipient teams, and the potential for prolonged cold ischemia times. Hypothermic machine perfusion, given the agility of the device and the ability to surgically manipulate the graft during a period of theoretical reconditioning, may help address some of these challenges by extending reasonable preservation time enabling safe and effective parenchymal transection.

A major advantage of performing ex situ liver splitting with HOPE is the reduction of cold ischemia and the prevention of uncontrolled graft warming during prolonged back‐table manipulation of ex situ splits. Cold ischemia time remains an important determinant of liver‐transplant outcomes; each additional minute required for parenchymal transection, dissection, reconstruction, and hemostasis prolongs ischemic preservation and may permit intermittent graft rewarming as the liver is manipulated [15, 16]. Such temperature transitions may exacerbate mitochondrial dysfunction and ischemia–reperfusion injury [17]. In contrast, continuous hypothermic oxygenated perfusion provides stable intravascular cooling and oxygen delivery throughout the split [12]. The HOPE‐Split pilot study demonstrated that ex situ splitting during perfusion reduced cold time despite a longer total ex vivo preservation period [13], while back‐table preparation under continuous portal‐vein oxygenated perfusion similarly shortened nonperfused back‐table time and overall cold ischemia. The demonstrated safety of prolonged hypothermic oxygenated perfusion suggests that time need no longer be the adversary it has traditionally been in split‐liver transplantation, easing the logistics associated with split liver grafts [18, 19]. Instead, continuous perfusion may allow unhurried graft splitting and reconstruction while limiting ischemia and manipulation‐associated rewarming.

There are a few technical considerations in this case that warrant further discussion and exploration. The protective effects of HOPE depend on the integrity of the Belzer MPS and progressive dilution with normal saline while using the CUSA could theoretically attenuate these benefits. While not used in this specific case, a pragmatic solution would be to use Belzer MPS itself as the CUSA irrigation fluid rather than saline, or to employ a drainage strategy that prevents saline from entering the recirculating perfusion circuit; however, none of the published HOPE‐Split studies have explicitly addressed this technical detail. This yet‐to‐be solved problem represents an important gap in the procedural standardization literature. Additionally, it is worth noting that despite the use of thermal devices to accomplish the transection, the temperature of the perfusate never went above 10 degrees centigrade. We used a thermal device to control cut‐surface bleeding at reperfusion, which is a known drawback of ex situ splits, while keeping temperatures within the 4°C–12°C range required for HOPE to be effective. This finding strengthens the argument that HOPE‐based ex situ splitting can mitigate the historically higher complication rates of split grafts and should be highlighted as a technical innovation that addresses a known criticism of the split technique.

This approach has several important limitations. Most notably, hypothermic oxygenated machine perfusion is not universally available and requires specialized equipment, trained perfusion personnel, institutional expertise, and protocols for graft cannulation, monitoring, transport, and troubleshooting. The acquisition and maintenance costs of the perfusion device, disposable circuits, preservation solution, and additional staffing may be substantial, particularly for lower‐volume transplant centers, and these expenses may not be consistently reimbursed. Performing the split while the liver remains on perfusion may also increase technical complexity and certainly requires additional operating‐room coordination when two recipient teams or institutions are involved. The ability to continue HOPE closer to implantation may represent an opportunity to further reduce non‐oxygenated cold preservation time when institutional logistics, recipient timing, and perfusion resources permit. However, as demonstrated in this case, these challenges can be overcome to incorporate hypothermic oxygenated machine perfusion as a novel strategy for ex situ graft splitting, ultimately creating an opportunity for transplantation in a pediatric patient that otherwise would not have been possible. Lastly, no biochemical viability testing of the perfusate was performed in this case. In particular, perfusate flavin mononucleotide (FMN) testing, which can be used as a biomarker of mitochondrial injury during hypothermic oxygenated perfusion, is not currently available at our center. Perfusion was monitored using the device parameters, including flow, pressure, and temperature, with subsequent assessment of graft function following reperfusion. Objective biomarkers such as FMN may provide additional information regarding graft viability in future applications of this technique.

## 4 Conclusion

High‐volume pediatric transplant centers with technical variant experience should consider splitting deceased donor liver allografts on hypothermic machine perfusion as a strategy to increase split graft utilization and expand access to liver transplantation in the United States.

## Funding

The authors have nothing to report.

## Conflicts of Interest

The authors declare no conflicts of interest.

## Data Availability

Research data are not shared.
